# TSPNet: a time-spatial parallel network for classification of EEG-based multiclass upper limb motor imagery BCI

**DOI:** 10.3389/fnins.2023.1303242

**Published:** 2023-12-15

**Authors:** Jingfeng Bi, Ming Chu, Gang Wang, Xiaoshan Gao

**Affiliations:** ^1^School of Automation, Beijing University of Posts and Telecommunications, Beijing, China; ^2^School of Automation Science and Electrical Engineering, Beihang University, Beijing, China

**Keywords:** brain-computer interface, deep learning, electroencephalogram, multi classification, motor imagery

## Abstract

The classification of electroencephalogram (EEG) motor imagery signals has emerged as a prominent research focus within the realm of brain-computer interfaces. Nevertheless, the conventional, limited categories (typically just two or four) offered by brain-computer interfaces fail to provide an extensive array of control modes. To address this challenge, we propose the Time-Spatial Parallel Network (TSPNet) for recognizing six distinct categories of upper limb motor imagery. Within TSPNet, temporal and spatial features are extracted separately, with the time dimension feature extractor and spatial dimension feature extractor performing their respective functions. Following this, the Time-Spatial Parallel Feature Extractor is employed to decouple the connection between temporal and spatial features, thus diminishing feature redundancy. The Time-Spatial Parallel Feature Extractor deploys a gating mechanism to optimize weight distribution and parallelize time-spatial features. Additionally, we introduce a feature visualization algorithm based on signal occlusion frequency to facilitate a qualitative analysis of TSPNet. In a six-category scenario, TSPNet achieved an accuracy of 49.1% ± 0.043 on our dataset and 49.7% ± 0.029 on a public dataset. Experimental results conclusively establish that TSPNet outperforms other deep learning methods in classifying data from these two datasets. Moreover, visualization results vividly illustrate that our proposed framework can generate distinctive classifier patterns for multiple categories of upper limb motor imagery, discerned through signals of varying frequencies. These findings underscore that, in comparison to other deep learning methods, TSPNet excels in intention recognition, which bears immense significance for non-invasive brain-computer interfaces.

## 1 Introduction

Brain-computer interface (BCI) plays a pivotal role in facilitating communication and control between the human brain and external devices (Ang and Guan, 2015; Chaudhary et al., 2016). Among various techniques, electroencephalography (EEG) offers a notable advantage in terms of its superior time resolution when compared to similar methods like functional magnetic resonance imaging and near-infrared spectroscopy. The enhanced temporal resolution of EEG enables swift communication between users and computers, which, in turn, contributes significantly to the development of rehabilitation systems for patients with tetraplegia and aids in supporting the daily activities of healthy individuals (Suk and Lee, 2013; Leeb et al., 2015; Ang and Guan, 2017). A multitude of algorithms have been developed for EEG pattern classification in diverse BCI applications (Iacoviello et al., 2016; Foong et al., 2020; Zhang et al., 2020; Wang et al., 2021, 2023; Chen et al., 2022; She et al., 2023). In their research, Wang et al. (2021) redefined the common spatial pattern (CSP) as a constrained minimization problem, establishing equivalence between the reformulated CSP and the original CSP. Additionally, Zhang et al. (2020) proposed a deep learning framework that incorporates convolutional and recurrent neural networks. EEG-based BCI applications commonly rely on four main types of neurophysiological patterns, namely, steady-state visual evoked potential (SSVEP) (Autthasan et al., 2020; Kwak and Lee, 2020; Rivera-Flor et al., 2022; Zhang et al., 2022; Chailloux Peguero et al., 2023; Yan et al., 2023), event-related potential (ERP) (Cecotti and Graeser, 2011; Zou et al., 2016; Li et al., 2020), movement-related cortical potentials (MRCPs) (Xu et al., 2014; Jeong et al., 2020), and motor imagery (MI) (Siuly and Li, 2012; Higashi and Tanaka, 2013; Edelman et al., 2016; He et al., 2016; Chaisaen et al., 2020; Wu et al., 2020; Gaur et al., 2021; Ma et al., 2022; Fan et al., 2023; Zhang et al., 2023). Among these EEG applications, MI has garnered increasing attention within BCI systems due to its ability to elicit oscillatory neural activity in specific frequency bands over the motor cortex region without external stimuli.

In previous research on MI, Duan et al. (2021) proposed a binary standard task-related component analysis method (bSTRCA). In bSTRCA, correlation coefficients were extracted as features, and a linear discriminant analysis classifier was then used to classify the features. Filter bank selection can further enhance the performance of bSTRCA, leading to the introduction of the binary filter bank task-related component analysis (bFBTRCA) method (Jia et al., 2022). Additionally, they adapted the structure of the bSTRCA method for multi-class standard task-related component analysis (mSTRCA). Moreover, the multi-class filter bank task-related component analysis (mFBTRCA) method (Jia et al., 2023) was developed by integrating filter bank selection into mSTRCA. This method is applied to classify multi-class limb movements by segmenting MRCP signals into low-frequency filter banks. It optimizes multi-channel signals within these banks using spatial filters to extract correlation features, which are then combined and classified using a support vector machine. Jin et al. (2020) introduced a sparse Bayesian ELM-based algorithm to enhance the classification performance of MI. Jin et al. (2019) proposed a correlation-based channel selection (CCS) method to identify channels that contain more correlated information. Zhang et al. (2019) introduced a novel algorithm called temporally constrained sparse group spatial pattern (TSGSP) for simultaneously optimizing filter bands and time windows within CSP to further improve the classification accuracy of MI EEG. Jiao et al. (2019) presented a novel sparse group representation model (SGRM) to enhance the efficiency of MI-based BCI by leveraging intrasubject information. Barachant et al. (2012) introduced a new classification framework that incorporates the concept of Riemannian geometry into the manifold of covariance matrices. Aghaei et al. (2016) proposed separable common spatial-spectral patterns (SCSSP). Most of the previous MI-based research has produced excellent results, but the current BCI system based on MI can only effectively distinguish between left and right motor execution/imagery.

Deep learning (DL), as a subcategory of machine learning, currently represents the state-of-the-art approach in computer vision and natural language processing applications (Sakhavi et al., 2018). Beyond its application in computer vision, DL has also found utility in various domains, including brain-computer interfaces (BCI). Recent findings by Schirrmeister et al. (2017) have demonstrated that advancements in machine learning, such as batch normalization and exponential linear units, when combined with a carefully curated training strategy, have significantly enhanced the performance of deep convolutional neural networks (DCNNs) in decoding, achieving results on par with the widely adopted filter bank common spatial patterns (FBCSP) algorithm. In a novel development, Vuckovic and Sepulveda (2012) introduced a two-modality, four-category BCI classifier based on motor imagery involving movements of the left and right wrists. Meanwhile, Hajinoroozi et al. (2016) put forward an innovative channel-wise convolutional neural network (CCNN) architecture. Additionally, they explored CCNN-R, a variant of CCNN employing restricted Boltzmann machines to replace conventional convolutional filters. Furthermore, Tabar and Halici (2017) conducted a study on the classification of EEG motor imagery signals using convolutional neural networks (CNNs) and stacked autoencoders (SAEs). They proposed a new deep network by amalgamating CNNs and SAEs. Despite notable advancements in recent years, limitations persist in motor imagery-based BCI research. The primary focus has been on binary classification tasks, such as distinguishing between left-hand and right-hand motor imagery tasks or right-hand and right-foot motor imagery tasks, among others. Related research has, at most, extended to four-category classification problems, such as distinguishing between left and right hand, foot, and tongue motor imagery tasks. In reality, human upper limb movements encompass six distinct and typical categories, including elbow flexion, elbow extension, forearm supination, forearm pronation, hand open, and hand close. These six classes encompass the natural and continuous spectrum of upper limb movements. However, existing EEG-based motor imagery classifications have been limited to just two or four categories. This limitation starkly contrasts with the way individuals naturally plan to execute movements, hindering the full replication and support of the richness and diversity of human upper limb actions.

In this paper, we introduce a Time-Spatial Parallel Network (TSPNet) based on deep learning for the classification of six categories of upper limb movements. The TSPNet comprises three critical components: the Time Dimension Feature Extractor (TDFE) and the Spatial Dimension Feature Extractor (SDFE) for extracting temporal and spatial features, and the Time-Spatial Parallel Feature Extractor (TSPFE) for parallelizing time-spatial features. Specifically, the TDFE module employs residual convolutional blocks to extract temporal features, while the SDFE module utilizes residual convolutional blocks to extract spatial features. The TSPFE module subsequently eliminates the correlation between temporal and spatial features to reduce feature redundancy. Furthermore, the TSPFE module utilizes a gating mechanism to optimize weight distribution and parallelize time-spatial features. Diverging from existing networks that employ binary classification, our deep learning model in this study adopts multi-class classification. Additionally, we propose a feature visualization algorithm based on signal occlusion frequency to qualitatively analyze the proposed TSPNet. In summary, the primary contributions of our work are as follows:

1. A time-spatial parallel network (TSPNet) is introduced for the recognition of six classes of upper limb motor imagery.
2. Within TSPNet, a critical module called TSPFE is introduced to parallelize time-spatial features.
3. We provide a publicly accessible dataset containing EEG data from ten individuals, comprising a total of 1,800 samples of upper limb motor imagery data (hand open, hand close, forearm supination, forearm pronation, elbow flexion, and elbow extension) categorized into six classes.

The remainder of this article is organized as follows. In Section 2, we offer a comprehensive exploration of the architecture of our proposed TSPNet model, along with a detailed description of the feature visualization algorithm based on signal occlusion frequency that we have put forth. Moving on to Section 3, we present the datasets and implementation details, accompanied by ablation studies and a thorough comparison of experimental results. Moreover, we conduct experiments related to feature visualization in this section. Finally, Section 4 provides the conclusion to this article.

## 2 Methods

In this section, we introduce the Time-Spatial Parallel Network (TSPNet). We provide a detailed description of its key components, namely the Time Dimension Feature Extractor (TDFE), Spatial Dimension Feature Extractor (SDFE), and Time-Spatial Parallel Feature Extractor (TSPFE). Furthermore, we present an algorithm for feature visualization based on occluded input signal frequency, which is used for qualitative analysis of TSPNet. Our code will be publicly available on “https://github.com/Special4519/TSPNet.”

### 2.1 Time-spatial parallel network framework

**Figure 1:**
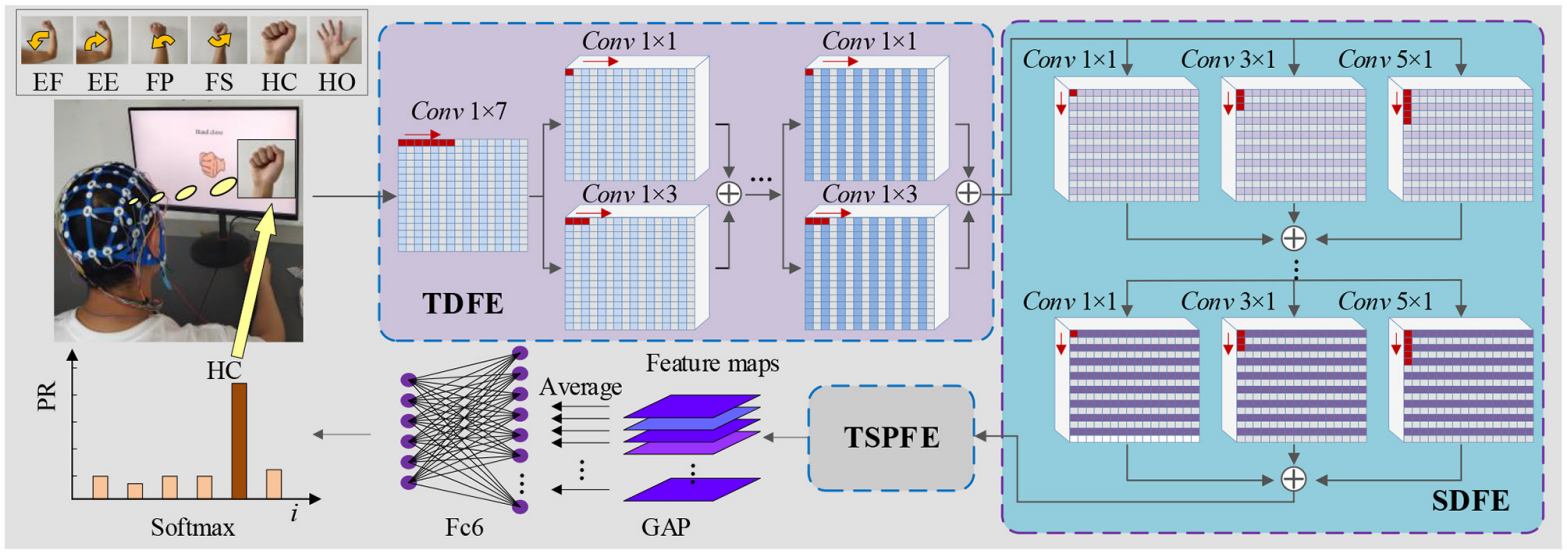
Architecture of TSPNet: mainly comprising three components—TDFE module, SDFE module, and TSPFE module.

As depicted in Figure 1, the proposed TSPNet comprises three main components: the Time Dimension Feature Extractor (TDFE), the Spatial Dimension Feature Extractor (SDFE), and the Time-Spatial Parallel Feature Extractor (TSPFE). Specifically, the TDFE employs a convolutional layer with kernel sizes of 1 × 7 to detect time dimension features from the input EEG signals. The structure of the input EEG is represented as [16, 1, 500, 1], where 16 denotes the number of signal channels, and 1,500 represents the sampling time points (the product of sampling frequency and time). Next, the TDFE uses residual convolutional blocks with kernel sizes of 1 × 1 and 1 × 3 in a parallel structure to extract shallow and deep time features within the time dimension. The resulting output is then fed into the SDFE. Let ***I***_EEG_ represent the original input EEG signals; this stage can be formulated as:


(1) $$\begin{array}{l} F_{\text{TD}}=H_{\text{TDFE}}\left(I_{\text{EEG}}\right)=H_{\text{HT}}\left(H_{\text{MT}}\left(H_{\text{LT}}\left(I_{\text{EEG}}\right)\right)\right) \end{array}$$

where *H*_TDFE_(·) represents the time dimension feature extraction procedure, which is divided into the shallow time feature extraction step *H*_LT_(·), the middle time feature extraction step *H*_MT_(·), and the deep time feature extraction step *H*_HT_(·). ***F***_TD_ is the output time dimension feature vector from the TDFE module.

The SDFE employs residual convolutional blocks with kernel sizes of 1 × 1, 3 × 1, and 5 × 1 in a parallel structure to extract spatial features. The input to this stage is ***F***_TD_, and the output is then fed into the TSPFE. This stage can be formulated as:


(2) $$\begin{array}{l} F_{\text{SD}}=H_{\text{SDFE}}\left(F_{\text{TD}}\right) \end{array}$$

where *H*_SDFE_(·) denotes the spatial dimension feature extraction procedure. ***F***_SD_ is the output spatial dimension feature vector from the SDFE module. The feature ***F***_SD_ extracted by the SDFE module is used as the input for the TSPFE module. First, the TSPFE removes the connection between time features and spatial features to eliminate redundancy. Then, the TSPFE employs a gating mechanism to achieve a more effective weight distribution and parallelize time-spatial features. Finally, the output ***F***_TSP_ is pooled by global average pooling (GAP) and connected to the fully connected layer and the softmax layer.

We adopt the generic cross-entropy loss function to train the proposed TSPNet model. defined as follows:


(3) $$\begin{array}{l} loss=-\frac{1}{N}\overset{N}{\underset{n=1}{\sum }}\overset{K}{\underset{i=1}{\sum }}w_{i}t_{ni}\ln\;y_{ni} \end{array}$$

where *N* is the number of samples, *K* is the number of categories, ***w***_*i*_ represents the weight for category *i*, ***t***_*ni*_ indicates whether the *n*th sample belongs to the *i*th category, and ***y***_*ni*_ is the output for sample *n* and category *i*, which is determined by the softmax function. ***y***_*ni*_ also represents the probability that the network associates the *n*th input with category *i*.

#### 2.1.1 Time dimension feature extractor

**Figure 2:**
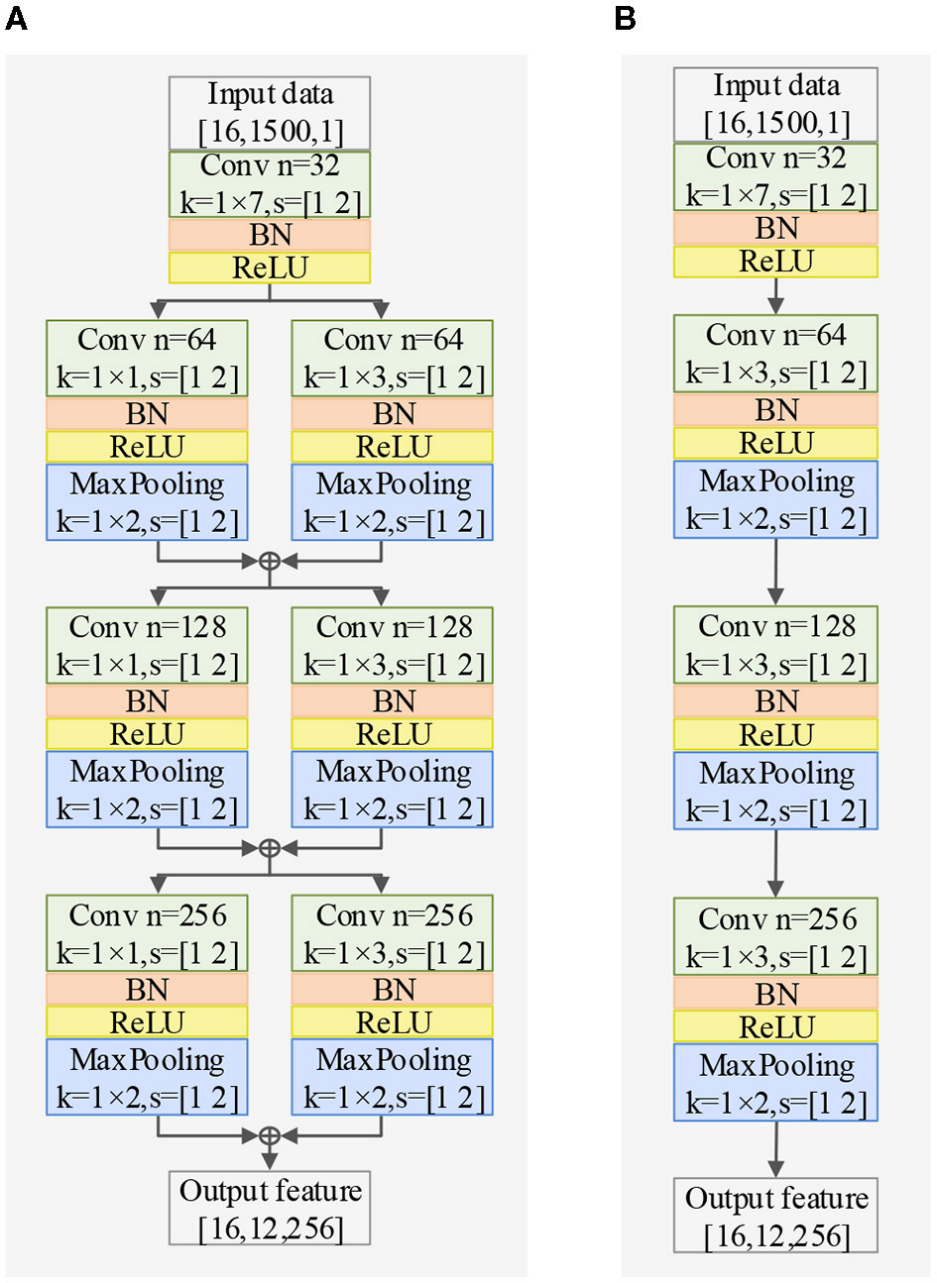
Exploring different TDFE forms: a comparative study of two structures in classification accuracy, demonstrating the superiority of the residual block TDFE, with BN representing batch normalization layer. **(A)** Structure of TDFE. **(B)** Structure of TDFE-NR.

The EEG signal is a type of non-stationary and nonlinear signal with strong randomness (Garcia-Martinez et al., 2021). Traditional signal processing methods are based on the theoretical analysis of linear systems, which inevitably results in the loss of a significant amount of information carried by the original signal. In order to extract complex features in the time dimension, we propose a Time Dimension Feature Extractor (TDFE) module that only convolves in the time dimension, as shown in Figure 2A. We also compare the TDFE with a non-residual block TDFE-NR, as shown in Figure 2B. The comparison results are presented in Section III. In the TDFE, we utilize a convolutional layer with kernel sizes of 1×7 to increase the receptive field of the network. This allows the TDFE to cover a larger area with the convolutional filters. Subsequently, the shallow time feature extraction step *H*_LT_(·) can be defined as


(4) $$\begin{array}{l} x_{\text{LT}}=\sigma \left[\Gamma \left(x_{\text{in}},w_{1\times 1}^{64}\right)\right]+\sigma \left[\Gamma \left(x_{\text{in}},w_{1\times 3}^{64}\right)\right] \end{array}$$

where, ***x***_LT_ represents the shallow time feature vector, ***x***_in_ is the input vector for the shallow time feature extraction step, σ denotes the ReLU activation function, Γ represents the residual mapping to be learned, and $w_{1\times 1}^{64}$ and $w_{1\times 3}^{64}$ are the weights of 1 × 1 and 1 × 3 convolutional kernels with 64 channels, respectively. The shallow time feature extraction step uses residual convolutional blocks with kernel sizes of 1×1 and 1×3 in parallel to fuse different-level features of the input ***x***_in_ into the shallow time feature vector. Similarly, the middle time feature extraction step *H*_MT_(·) and the deep time feature extraction step *H*_HT_(·) can be defined as


(5) $$\begin{array}{l} x_{\text{MT}}=\sigma \left[\Gamma \left(x_{\text{LT}},w_{1\times 1}^{128}\right)\right]+\sigma \left[\Gamma \left(x_{\text{LT}},w_{1\times 3}^{128}\right)\right] \end{array}$$

(6) $$\begin{array}{l} x_{\text{HT}}=\sigma \left[\Gamma \left(x_{\text{MT}},w_{1\times 1}^{256}\right)\right]+\sigma \left[\Gamma \left(x_{\text{MT}},w_{1\times 3}^{256}\right)\right] \end{array}$$

where ***x***_MT_ represents the middle time feature vector, ***x***_HT_ represents the deep time feature vector, and it is also the time dimension output feature vector of the TDFE module.

#### 2.1.2 Spatial dimension feature extractor

**Figure 3:**
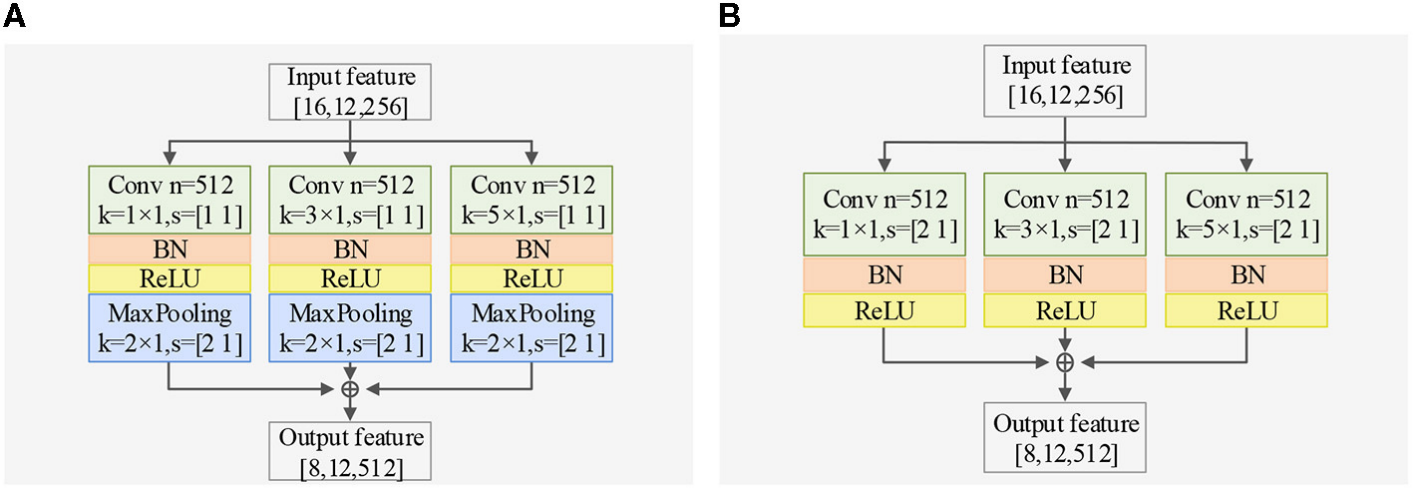
Exploring different SDFE forms: a comparative study of two structures in classification accuracy, demonstrating the superiority of SDFE, with BN representing batch normalization layer. **(A)** Structure of SDFE. **(B)** Structure of SDFE-NP.

Regarding spatial dimension feature extraction, we introduce two spatial feature extractors. The Spatial Dimension Feature Extractor (SDFE) can be seen in Figure 3A and utilizes a max-pooling layer to reduce the size of the feature map. On the other hand, SDFE-NP, shown in Figure 3B, omits the max-pooling layer but sets the convolution stride in the spatial dimension to 2. In terms of convolutional structure, both SDFE and SDFE-NP employ three groups of parallel structures with convolutional kernels of different sizes to extract spatial dimension features at various levels. This stage can be expressed as follows:


(7) $$\begin{array}{l} F_{\text{SD}}=\underset{\beta =1,3,5}{\sum }\sigma \left[\Gamma \left(x_{\text{HT}},w_{\beta \times 1}^{512}\right)\right] \end{array}$$

where, ***F***_SD_ represents the spatial dimension output feature vector, β is the number of residual paths, σ denotes the ReLU activation function, Γ signifies the residual mapping to be learned, and $w_{1\times 1}^{512}$, $w_{3\times 1}^{512}$, and $w_{5\times 1}^{512}$ are the weights of 1 × 1, 3 × 1, and 5 × 1 convolutional kernels, each with 512 channels.

#### 2.1.3 Time-spatial parallel feature extractor

**Figure 4:**
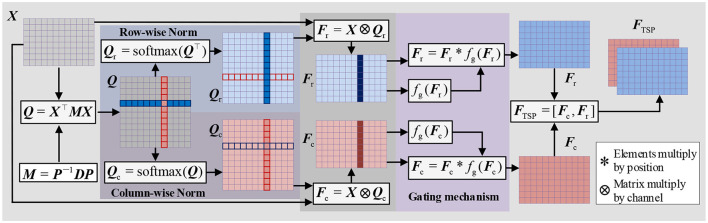
Structure of TSPFE.

To extract parallel features from both the time and spatial dimensions, we propose a Time-Spatial Parallel Feature Extractor (TSPFE), as illustrated in Figure 4. In the TSPFE, each channel's input data is processed separately and represented as ***X***. First, we calculate the affine transformation matrix ***Q*** ∈ ***R***^*W* × *W*^ through ***X*** ∈ ***R***^*H* × W^and transpose matrix ***X***^⊤^:


(8) $$\begin{array}{l} Q=X^{⊤}MX \end{array}$$

where, ***M*** ∈ ℝ^*H*×*H*^ represents a weight matrix. The elements of ***Q*** reflect the similarity between the time dimension and spatial dimension features. As ***M*** is a square matrix, its diagonalization can be expressed as:


(9) $$\begin{array}{l} M=P^{-1}DP \end{array}$$

where ***P*** is an invertible matrix, and ***D*** is a diagonal matrix. Subsequently, Eq. (8) can be rewritten as:


(10) $$\begin{array}{l} Q=X^{⊤}P^{-1}DPX \end{array}$$

Let ***M*** be a symmetric matrix; then ***M*** must be both orthogonal and diagonal. The orthogonal matrix ***P*** projects the feature into an orthogonal space, eliminating the connection between the time feature and the spatial feature to prevent redundancy. This stage can be formulated as:


(11) $$\begin{array}{l} Q=X^{⊤}P^{⊤}DPX={\left(PX\right)}^{⊤}DPX \end{array}$$

next, we normalize the columns and rows of the ***Q*** matrix and multiply it with the original ***X*** matrix:


(12) $$\begin{array}{l} Q_{\text{c}}=\text{softmax}\left(Q\right) \end{array}$$

(13) $$\begin{array}{l} Q_{\text{r}}=\text{softmax}\left(Q^{⊤}\right) \end{array}$$

(14) $$\begin{array}{l} F_{\text{c}}=X⊗Q_{\text{c}} \end{array}$$

(15) $$\begin{array}{l} F_{\text{r}}=X⊗Q_{\text{r}} \end{array}$$

where ⊗ denotes matrix multiplication by channel. ***F***_c_ and ***F***_r_ represent time features and spatial features, respectively. Considering that different channels and time points have varying importance, we introduce a gating mechanism to achieve better weight distribution:


(16) $$\begin{array}{l} F_{\text{c}}=F_{\text{c}}*f_{\text{g}}\left(F_{\text{c}}\right)=F_{\text{c}}*\sigma \left(w_{f}F_{\text{c}}+b_{f}\right) \end{array}$$

(17) $$\begin{array}{l} F_{\text{r}}=F_{\text{r}}*f_{\text{g}}\left(F_{\text{r}}\right)=F_{\text{r}}*\sigma \left(w_{f}F_{\text{r}}+b_{f}\right) \end{array}$$

where * denotes element-wise multiplication, σ denotes the ReLU activation function, ***w***_*f*_ represents the convolution weights, and *b*_*f*_ is the convolution bias. Finally, we combine ***F***_c_ and ***F***_r_ to obtain ***F***_TSP_, which is the time-spatial parallel feature:


(18) $$\begin{array}{l} F_{\text{TSP}}=\left[F_{\text{c}},F_{\text{r}}\right] \end{array}$$

The final extracted time-spatial parallel feature is pooled using global average pooling (GAP) and connected to the fully connected layer and the softmax layer.

### 2.2 Feature visualization algorithm

To qualitatively analyze TSPNet, we propose a feature visualization algorithm based on signal occlusion frequency, as illustrated in Algorithm 1. The test dataset is denoted as *T* = {*X*^1^, …, *X*^*M*^}. The real labels of the test dataset are denoted as *Y* = {*y*^1^, …, *y*^*M*^}, where *M* represents the total number of test trials. *f*(*X*, ω) is a well-trained TSPNet classifier, where ω represents the classifier's parameters. First, the test dataset *T* is input into the classifier to obtain the predicted labels *Y*_*p*_.

**Algorithm 1:** Feature visualization algorithm based on signal occlusion frequency.

**Input**: Test datasets *T* = {*X*^1^, …, *X*^*M*^}, real label of test datasets *Y* = {*y*^1^, …*y*^*M*^}, well-trained TSPNet classifier *f*(*X*, ω) with parameters ω, feature extraction function $\phi \left(X^{j},\omega_{\phi }\right)$ with parameters ω_ϕ_.
**Output**: Scalp topographic maps for different categories and different frequencies.
**Step 1**: Use the well-trained TSPNet classifier *f*(*X*, ω) with parameters ω to predict the label *Y*_*p*_ for the test datasets *T*.
*Y*_*p*_ = *f*(*T*, ω)
**Step 2**: Compare the predicted label *Y*_*p*_ with the real label *Y* to get the correctly recognized test datasets *T*_*c*_.
**Step 3**: Filter the correctly recognized test datasets *T*_*c*_ using the filters with frequency ranges (δ:0.5 − 3Hz, θ:3 − 7Hz, α:7 − 13Hz, β:13 − 200Hz) to obtain the filtered datasets *T*^δ^, *T*^θ^, *T*^α^, and *T*^β^ using Eq. (20).
**Step 4**: Extract the activated features for each filtered datasets *T*^δ^, *T*^θ^, *T*^α^, and *T*^β^ using the feature extraction function $\phi \left(X^{j},\omega_{\phi }\right)$ with parameters ω_ϕ_ to obtain the feature maps *F*^δ^, *F*^θ^, *F*^α^, and *F*^β^ using Eq. (21).
**Step 5**: Average the feature maps *F*^δ^, *F*^θ^, *F*^α^, and *F*^β^ according to different categories.
**Step 6**: Draw scalp topographic maps for different categories and different frequencies using the averaged feature maps from Step 5.


(19) $$\begin{array}{l} Y_{p}=f\left(T,\omega \right) \end{array}$$

We then compare the predicted labels *Y*_*p*_ with the real labels *Y* to identify the correctly recognized test dataset, denoted as *T*_*c*_. Next, *T*_*c*_ is filtered using filters with frequency ranges (δ:0.5 − 3Hz, θ:3 − 7Hz, α:7 − 13Hz, β:13 − 200Hz), expressed as


(20) $$\{\begin{array}{l} T^{\delta }=filter(T_{C},\delta )\\ T^{\theta }=filter(T_{C},\theta )\\ T^{\alpha }=filter(T_{C},\alpha )\\ T^{\beta }=filter(T_{C},\beta ) \end{array}$$

where, *T*^δ^, *T*^θ^, *T*^α^, and *T*^β^ represent the correctly recognized test datasets in different frequency ranges. These test datasets in different frequency ranges are then input into the feature extraction function $\phi \left(X^{j},\omega_{\phi }\right)$ of TSPNet to obtain the activated features in different frequency ranges, expressed as


(21) $$\{\begin{array}{l} F^{\delta }=\phi (T^{\delta },\omega_{\phi })\\ F^{\theta }=\phi (T^{\theta },\omega_{\phi })\\ F^{\alpha }=\phi (T^{\alpha },\omega_{\phi })\\ F^{\beta }=\phi (T^{\beta },\omega_{\phi }) \end{array}$$

The activated features in different frequency ranges are then averaged based on different categories. Finally, scalp topographic maps are generated for different categories and different frequencies.

## 3 Experiments and results

In this section, we begin by providing a brief overview of the datasets and our experimental setup. Following this, we conduct ablation studies. Subsequently, we compare TSPNet with various deep learning methods [MSATNet (Hu et al., 2023), EEGSym (Perez-Velasco et al., 2022), DeepConvNet (Schirrmeister et al., 2017), EEGNet-8,2 (Lawhern et al., 2018)] using two datasets. Finally, we perform experiments related to feature visualization.

### 3.1 Datasets

Dataset I was collected through our experiments. We recruited 10 healthy participants aged between 24 and 38 years, with a mean age of 30 years (standard deviation 5 years). Five of the participants are male, and all are right-handed. The study was conducted in accordance with the Declaration of Helsinki, and informed consent was obtained from all subjects. This study does not require ethical approval because of its non-invasive nature, utilization of anonymous data, and adherence to the Helsinki Declaration. Subjects had normal or corrected-to-normal vision and no history of neurological or psychiatric disorders. They performed six categories of motor imagery tasks involving elbow flexion, elbow extension, forearm supination, forearm pronation, hand open, and hand close, all related to the right upper limb. EEG signals were recorded using 16 active Ag/AgCl electrodes with the OpenBCI CytonDaisy 16-channel Biosensing Board. We applied an 8th order Chebyshev bandpass filter from 0.01 to 200 Hz and used a notch filter at 50 Hz to suppress power line interference. The sampling frequency was set to 500 Hz, with the reference electrode placed on the left earlobe and the ground on the right earlobe. EEG electrodes were positioned following the international standard 10–20 electrode system. Dataset I comprises 18,000 epochs (300 trials × six categories × 10 subjects). This dataset has been uploaded to IEEE DataPort and can be accessed at “https://dx.doi.org/10.21227/8qw6-f578.”

Dataset II is provided by Ofner et al. (2017) and is available in the BNCI Horizon 2020 database at “http://bnci-horizon-2020.eu/database/data-sets.” It includes electroencephalography (EEG) data from 15 healthy subjects aged between 22 and 40 years, with a mean age of 27 years (standard deviation 5 years). EEG signals were measured using 61 channels covering frontal, central, parietal, and temporal areas, employing active electrodes and four 16-channel amplifiers from g.tec medical engineering GmbH, Austria. In total, Dataset II contains 5,400 epochs (60 trials × six categories × 15 subjects).

### 3.2 Implementation details

In Dataset I and Dataset II, we train the data for each subject separately. In each training iteration, the data is divided into a training dataset and a testing dataset with a partition ratio of 70%–30%. The dataset is randomly shuffled, resulting in a total of 10 partitions. The average classification accuracy of these 10 experiments serves as the evaluation criterion. We employ the ADAM optimizer (Kingma and Ba, 2015) for model training, and the optimizer parameters are detailed in Table 1. The development of TSPNet is carried out using MATLAB R2020b (The MathWorks, Inc., Natick, MA, USA), and training is performed on a high-performance GPU (GeForce RTX 5000) integrated into an Intel (R) Core (TM) i7-7000K CPU processor with 64 GB RAM. For comparison, we evaluate TSPNet alongside other end-to-end deep learning methods, including MSATNet (Hu et al., 2023), EEGSym (Perez-Velasco et al., 2022), DeepConvNet (Schirrmeister et al., 2017), and EEGNet-8,2 (Lawhern et al., 2018). These methods are based on convolutional neural networks for EEG signal classification. To adapt these models to our datasets, we modify the classification number of the output layer to six, as required by the two datasets used in this study. Originally designed for EEG signals of 128 and 250 Hz, we down-sample the EEG signals in Dataset I and Dataset II to match their respective architectures. Training these models follows the same procedure as that of the TSPNet model.

**Table 1:** ADAM optimizer parameters.

**Parameters**	**Value**	**Parameters**	**Value**
Initial learn rate	0.001	Learn rate drop factor	0.1
Gradient threshold	1	Learn rate drop period	500
Max epochs	1,000	Squared gradient decay factor	0.999
Mini batch size	64	Gradient decay factor	0.9

### 3.3 Ablation studies on the Dataset I

In this section, we evaluate the impact of the proposed TDFE, SDFE, and TSPFE modules on the performance of TSPNet. Additionally, we validate the influence of different structures within the TDFE (Figure 2B) and SDFE (Figure 3B) modules on TSPNet. The experiments were conducted on Dataset I. Consistent with the details outlined in the implementation, during each ablation experiment, the training set and test set maintained a 70%–30% ratio, ensuring equal and balanced numbers for all classes to guarantee an equal chance level for each class. The experimental results are presented in Table 2, and a detailed analysis is provided below.

**Table 2:** Performance (mean ± SD) (in %) of ablation studies for TDFE, SDFE, and TSPFE on Dataset I.

**Subjects**	**Methods**					
	**TSPNet-w/o-TDFE**	**TSPNet-TDFE-NR**	**TSPNet-w/o-SDFE**	**TSPNet-SDFE-NP**	**TSPNet-w/o-TSPFE**	**TSPNet**
S1	25.8 ± 0.18	33.8 ± 0.17	24.6 ± 0.09	32.4 ± 0.13	22.3 ± 0.17	46.6 ± 0.11
S2	26.1 ± 0.16	31.2 ± 0.12	24.3 ± 0.11	33.6 ± 0.21	21.8 ± 0.08	42.4 ± 0.05
S3	28.9 ± 0.11	37.6 ± 0.14	27.2 ± 0.17	36.8 ± 0.11	25.4 ± 0.04	58.1 ± 0.03
S4	25.3 ± 0.07	32.5 ± 0.07	28.5 ± 0.12	31.8 ± 0.14	24.1 ± 0.07	51.5 ± 0.09
S5	23.5 ± 0.12	31.5 ± 0.08	25.9 ± 0.07	32.3 ± 0.08	23.4 ± 0.15	45.9 ± 0.07
S6	27.1 ± 0.10	30.3 ± 0.04	28.3 ± 0.08	35.4 ± 0.06	24.2 ± 0.03	48.6 ± 0.05
S7	25.6 ± 0.16	35.9 ± 0.18	24.1 ± 0.14	36.7 ± 0.07	25.8 ± 0.12	49.2 ± 0.13
S8	28.1 ± 0.19	33.5 ± 0.10	26.4 ± 0.13	31.4 ± 0.14	24.3 ± 0.08	48.2 ± 0.08
S9	32.5 ± 0.21	36.8 ± 0.07	31.3 ± 0.08	37.2 ± 0.11	31.8 ± 0.17	54.1 ± 0.05
S10	25.1 ± 0.07	30.8 ± 0.16	25.4 ± 0.20	31.4 ± 0.08	24.9 ± 0.14	46.4 ± 0.04
Mean	26.8 ± 0.024	33.4 ± 0.025	26.6 ± 0.022	33.9 ± 0.023	24.8 ± 0.026	**49.1** ± 0.043

Bold values represent the best results.

1) *Ablation studies for TDFE*: To demonstrate the effectiveness of the TDFE module, we remove the TDFE module and refer to it as TSPNet-w/o-TDFE. As shown in Table 2, when compared to TSPNet-w/o-TDFE, TSPNet exhibits a 22.3% increase in mean classification accuracy, indicating that the TDFE module, convolved in the time dimension, is effective for TSPNet. Furthermore, we replace the TDFE module with the non-residual block TDFE (referred to as TSPNet-TDFE-NR) to demonstrate the effectiveness of the residual block structure in TDFE. TSPNet shows improvement in all subjects, with a notable 20.5% boost in subject-3 (58.1% vs. 37.6%).

2) *Ablation studies for SDFE*: First, we validate the effectiveness of the SDFE module. We remove the SDFE module and refer to it as TSPNet-w/o-SDFE. Our TSPNet shows an overall improvement of 22.5% (49.1% vs. 26.6%), clearly demonstrating that the SDFE module, convolved in the spatial dimension, significantly enhances TSPNet's performance. Next, we replace the SDFE module with the non-maxpooling block SDFE (referred to as TSPNet-SDFE-NP) to illustrate the impact of the maxpooling structure in SDFE. When comparing TSPNet with TSPNet-SDFE-NP, TSPNet achieves an overall increment of 15.2% (49.1% vs. 33.9%).

3) *Ablation studies for TSPFE*: To further demonstrate the effectiveness of the TSPFE module, we remove the TSPFE module and denote it as TSPNet-w/o-TSPFE. Compared to TSPNet-w/o-TSPFE, TSPNet's mean classification accuracy increases by 24.3% (49.1% vs. 24.8%), underscoring the critical role of the TSPFE module in enhancing TSPNet.

### 3.4 Comparisons with the deep learning reference methods

**Figure 5:**
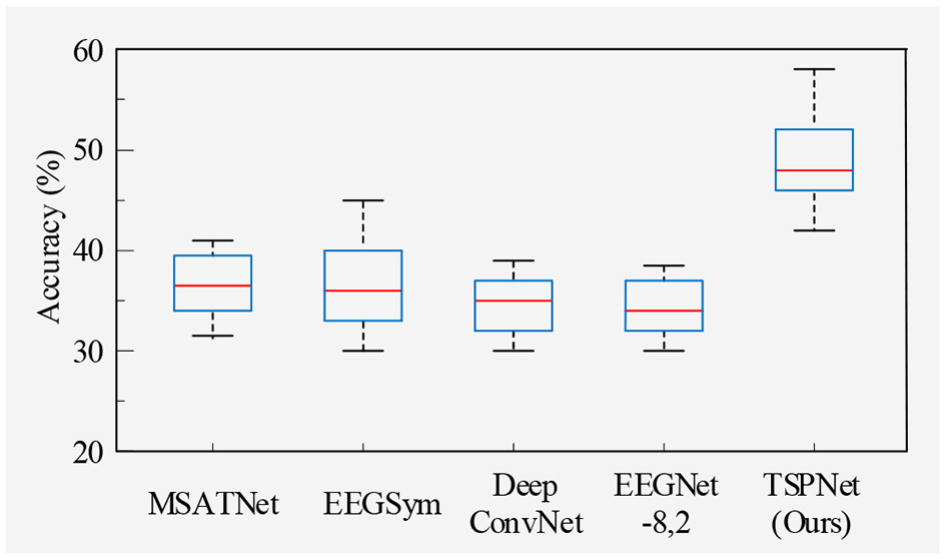
Box plot of classification accuracy for Dataset I.

1) *Quantitative analysis on the Dataset I*: TSPNet is compared with deep learning methods [MSATNet (Hu et al., 2023), EEGSym (Perez-Velasco et al., 2022), DeepConvNet (Schirrmeister et al., 2017), EEGNet-8,2 (Lawhern et al., 2018)]. The experimental results shown in Table 3 and Figure 5 demonstrate that TSPNet achieves the best mean accuracy on Dataset I. Compared to EEGNet-8,2, our TSPNet achieves approximately a 14.7% improvement in mean classification accuracy (49.1% vs. 34.4%). Notably, for subject-9, TSPNet outperforms EEGNet-8,2 by ~22.2% (54.1% vs. 31.9%). Because of the equal and balanced distribution of each class in the training data during the training process, the chance level for all six classification experiments in this study is 16.67%. To assess whether there is a significant difference in accuracy between TSPNet and other comparison methods, two-sample *t*-test was conducted in this study. The null hypothesis assumes that the accuracy of TSPNet and other comparison methods follows a normal distribution with equal means and unknown but identical variances. The alternative hypothesis is that there's a notable difference in accuracy between TSPNet and the other comparative methods. If the *p*-value is less than the significance level of 0.05, then the null hypothesis is rejected. The results, as indicated by the *p*-value in Table 3, signify a significant difference in accuracy between TSPNet and the other comparison methods.

**Table 3:** Performance (mean ± SD (in %) comparison with deep learning methods on Dataset I.

**Subjects**	**Methods**				
	**MSATNet**	**EEGSym**	**DeepConvNet**	**EEGNet-8,2**	**TSPNet (ours)**
S1	35.5 ± 0.21	38.6 ± 0.07	36.6 ± 0.04	36.9 ± 0.15	46.6 ± 0.11
S2	39.4 ± 0.15	39.2 ± 0.15	37.7 ± 0.07	33.4 ± 0.07	42.4 ± 0.05
S3	41.1 ± 0.16	44.5 ± 0.08	38.6 ± 0.21	38.4 ± 0.08	58.1 ± 0.03
S4	36.4 ± 0.12	31.5 ± 0.23	33.8 ± 0.11	30.4 ± 0.13	51.5 ± 0.09
S5	39.3 ± 0.07	33.8 ± 0.05	32.1 ± 0.06	30.6 ± 0.05	45.9 ± 0.07
S6	34.2 ± 0.13	39.3 ± 0.12	30.5 ± 0.09	31.8 ± 0.11	48.6 ± 0.05
S7	31.7 ± 0.22	33.8 ± 0.11	36.1 ± 0.12	36.8 ± 0.05	49.2 ± 0.13
S8	36.8 ± 0.11	39.3 ± 0.08	31.6 ± 0.18	38.2 ± 0.09	48.2 ± 0.08
S9	33.2 ± 0.07	33.6 ± 0.06	32.5 ± 0.07	31.9 ± 0.06	54.1 ± 0.05
S10	37.4 ± 0.05	30.4 ± 0.14	36.5 ± 0.04	35.6 ± 0.17	46.4 ± 0.04
Mean	36.5 ± 0.028	36.4 ± 0.042	34.6 ± 0.027	34.4 ± 0.030	**49.1** ± 0.043
*p*-value	4.46*e*^−5^	4.53*e*^−5^	1.00*e*^−5^	6.79*e*^−6^	–

Bold values represent the best results.

**Figure 6:**
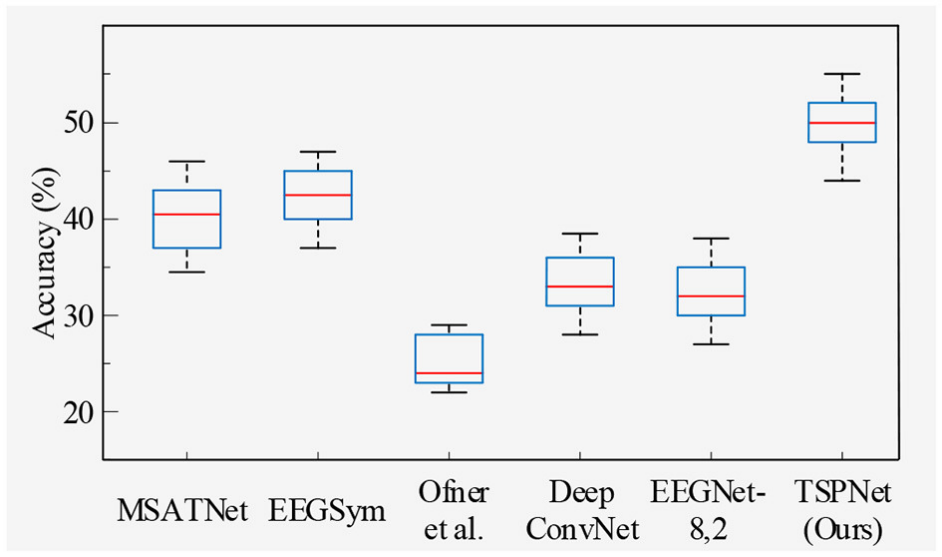
Box plot of classification accuracy for data from 61 channels in Dataset II.

**Figure 7:**
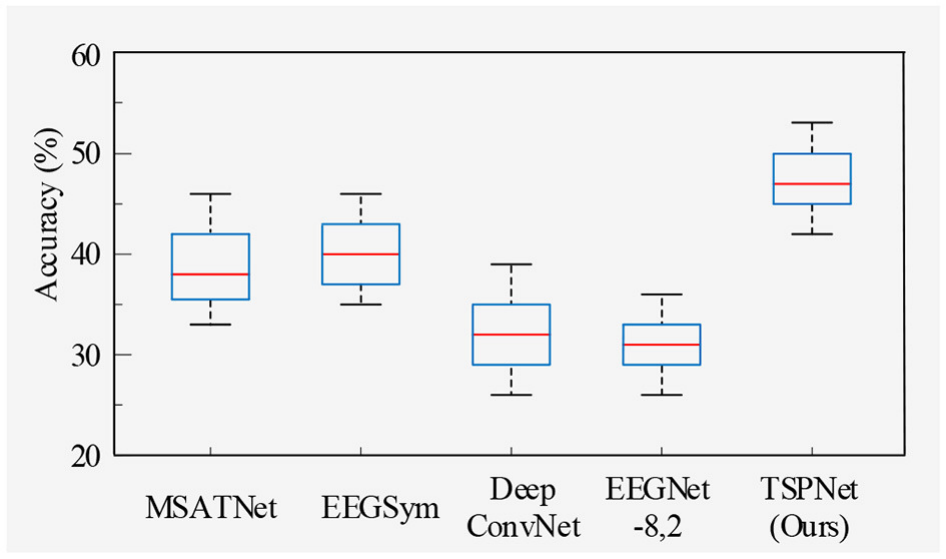
Box plot of classification accuracy for data from 16 channels in Dataset II.

2) *Quantitative analysis on the Dataset II*: We evaluate the proposed TSPNet on Dataset II to demonstrate its advantages. First, we use all 61-channel EEG signals in Dataset II for experiments. The classification accuracy experimental results of 15 subjects are listed in Table 4. It can be seen from Table 4 and Figure 6 that our TSPNet achieves an average classification accuracy of 49.7 ± 0.029, which is superior to all other comparison methods. Compared with Ofner et al. (2017), the performance of TSPNet has improved, with a relative improvement of 24.5% (49.7% vs. 25.2%). Compared with EEGNet-8,2, the performance of TSPNet has improved, with a relative improvement of 17.5% (49.7% vs. 32.2%). Furthermore, to compare the influence of EEG signals with different channel numbers on classification results, we select 16-channel EEG signals corresponding to Dataset I from Dataset II for classification experiments. As shown in Table 5 and Figure 7, TSPNet also achieves the best mean accuracy. As can be seen from Tables 4, 5, the mean classification accuracy of 61-channel data is 2.2% (49.7% vs. 47.5%) higher than that of 16-channel data. One possible reason is that more channels contain more spatial information. The *p*-values of two-sample *t*-test for TSPNet and other comparison methods, as shown in Tables 4, 5, indicate a significant difference in classification accuracy between TSPNet and the other comparison methods on Dataset II.

**Table 4:** Performance (mean ± SD) (in %) comparison with deep learning methods on Dataset II using 61 channels.

**Subjects**	**Methods**					
	**MSATNet**	**EEGSym**	**Ofner et al**.	**DeepConvNet**	**EEGNet-8,2**	**TSPNet (Ours)**
S1	40.4 ± 0.03	46.1 ± 0.04	29	37.2 ± 0.03	32.1 ± 0.05	49.7 ± 0.03
S2	35.4 ± 0.04	39.4 ± 0.07	23	38.5 ± 0.07	35.1 ± 0.13	48.6 ± 0.13
S3	45.7 ± 0.12	47.2 ± 0.03	23	36.1 ± 0.11	37.8 ± 0.12	53.2 ± 0.05
S4	38.7 ± 0.07	41.7 ± 0.11	29	36.1 ± 0.04	35.7 ± 0.03	49.4 ± 0.12
S5	36.3 ± 0.04	41.9 ± 0.08	24	32.8 ± 0.06	29.5 ± 0.06	48.9 ± 0.06
S6	34.4 ± 0.11	43.2 ± 0.12	24	31.4 ± 0.03	31.6 ± 0.13	45.9 ± 0.07
S7	44.3 ± 0.08	36.8 ± 0.13	24	30.8 ± 0.07	31.2 ± 0.08	52.8 ± 0.04
S8	35.7 ± 0.12	37.9 ± 0.07	26	33.9 ± 0.05	29.7 ± 0.11	52.5 ± 0.13
S9	43.5 ± 0.06	42.5 ± 0.12	28	32.4 ± 0.08	32.5 ± 0.05	54.8 ± 0.12
S10	39.4 ± 0.14	45.4 ± 0.05	23	30.5 ± 0.13	27.5 ± 0.11	47.4 ± 0.07
S11	42.6 ± 0.04	45.8 ± 0.11	22	33.4 ± 0.03	28.5 ± 0.04	44.5 ± 0.09
S12	38.5 ± 0.13	43.2 ± 0.08	28	28.6 ± 0.09	32.1 ± 0.13	45.8 ± 0.05
S13	42.4 ± 0.08	43.7 ± 0.06	27	34.4 ± 0.06	30.5 ± 0.03	50.9 ± 0.04
S14	41.6 ± 0.06	37.9 ± 0.09	25	31.5 ± 0.13	35.4 ± 0.12	49.8 ± 0.07
S15	46.2 ± 0.03	41.2 ± 0.04	23	35.2 ± 0.04	33.5 ± 0.06	50.7 ± 0.03
Mean	40.3 ± 0.037	42.3 ± 0.031	25.2 ± 0.023	33.5 ± 0.027	32.2 ± 0.028	**49.7** ± 0.029
*p*-value	9.33*e*^−8^	4.78*e*^−5^	1.03*e*^−13^	5.32*e*^−11^	9.38*e*^−12^	–

Bold values represent the best results.

**Table 5:** Performance (mean ± SD) (in %) comparison with deep learning methods on Dataset II using 16 channels.

**Subjects**	**Methods**				
	**MSATNet**	**EEGSym**	**DeepConvNet**	**EEGNet-8,2**	**TSPNet (Ours)**
S1	34.3 ± 0.04	37.4 ± 0.12	29.9 ± 0.06	29.9 ± 0.12	51.3 ± 0.08
S2	33.1 ± 0.07	37.4 ± 0.05	33.7 ± 0.13	33.2 ± 0.08	46.2 ± 0.05
S3	45.4 ± 0.03	45.4 ± 0.13	38.7 ± 0.12	35.8 ± 0.07	53.1 ± 0.04
S4	35.9 ± 0.12	34.8 ± 0.08	31.8 ± 0.05	28.6 ± 0.14	44.3 ± 0.04
S5	36.1 ± 0.04	37.5 ± 0.08	30.3 ± 0.08	31.1 ± 0.06	42.2 ± 0.03
S6	35.8 ± 0.07	40.4 ± 0.12	35.5 ± 0.04	35.1 ± 0.07	47.9 ± 0.08
S7	42.1 ± 0.06	42.6 ± 0.03	29.4 ± 0.05	26.6 ± 0.09	45.6 ± 0.06
S8	35.5 ± 0.11	38.9 ± 0.13	37.8 ± 0.12	30.6 ± 0.07	43.8 ± 0.12
S9	43.5 ± 0.13	42.6 ± 0.04	26.5 ± 0.13	31.2 ± 0.04	47.2 ± 0.07
S10	42.4 ± 0.04	43.4 ± 0.09	30.9 ± 0.06	28.8 ± 0.13	45.4 ± 0.02
S11	41.7 ± 0.04	43.8 ± 0.13	28.6 ± 0.07	30.1 ± 0.12	47.9 ± 0.03
S12	38.4 ± 0.13	38.6 ± 0.04	35.6 ± 0.13	29.1 ± 0.04	51.2 ± 0.07
S13	34.3 ± 0.05	35.2 ± 0.04	36.4 ± 0.08	34.6 ± 0.05	52.7 ± 0.13
S14	39.8 ± 0.07	43.4 ± 0.06	34.7 ± 0.07	33.5 ± 0.08	48.3 ± 0.03
S15	39.5 ± 0.13	42.8 ± 0.07	33.7 ± 0.09	32.8 ± 0.13	44.8 ± 0.08
Mean	38.5 ± 0.037	40.3 ± 0.033	32.9 ± 0.035	31.4 ± 0.026	**47.5** ± 0.032
*p*-value	4.29*e*^−6^	2.86*e*^−5^	1.03*e*^−9^	2.53*e*^−11^	–

Bold values represent the best results.

### 3.5 Visualization experiments

#### 3.5.1 Visualization of EEG source estimation

**Figure 8:**
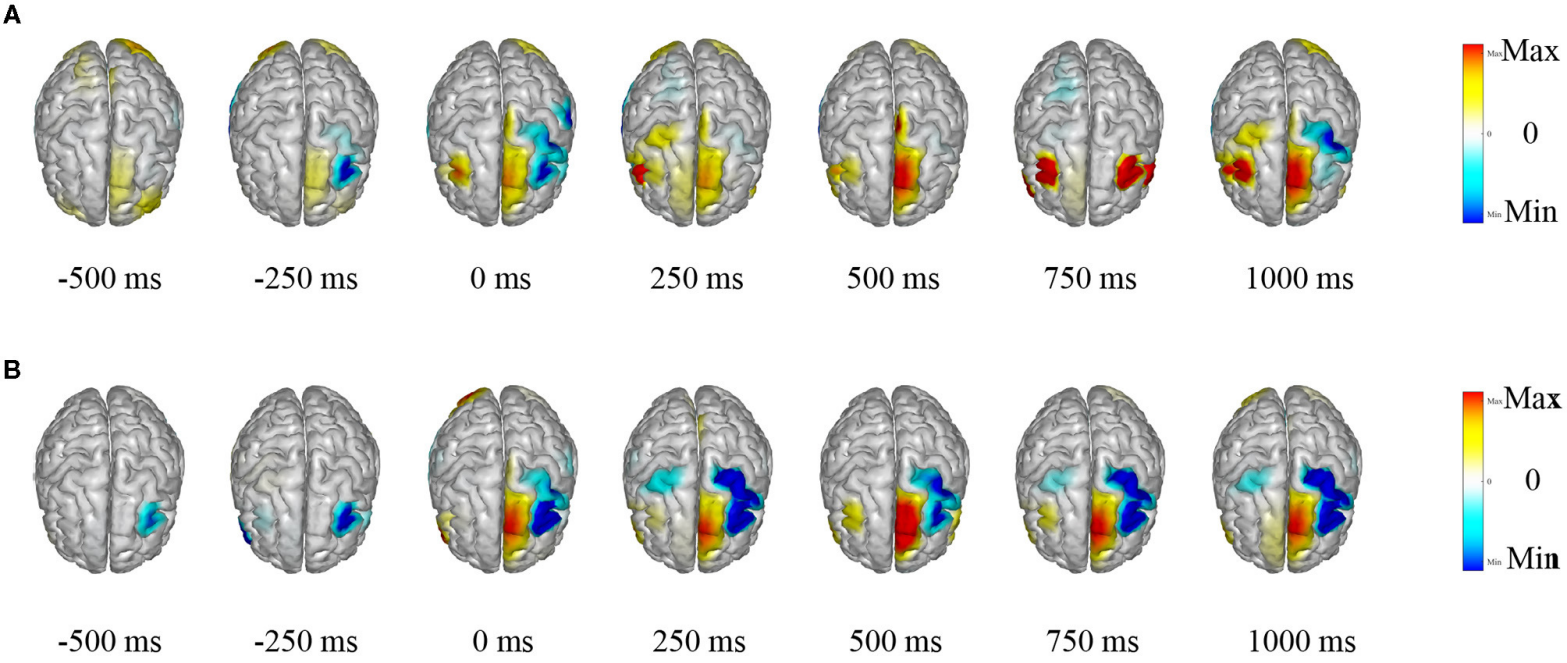
Visualization of EEG source estimation. **(A)** Visualization of EEG source estimation for Dataset I. **(B)** Visualization of EEG source estimation for Dataset II.

The TSPNet, as proposed in this article, is a brain-computer interface (BCI) model based on motor imagery (MI). The underlying principle of the MI-BCI system is that when a person envisions a movement, specific regions of their brain become activated, leading to alterations in their EEG signals. LORETA (Pascual-Marqui et al., 1994) is employed to visualize the source estimation of EEG data for the two datasets utilized in this article. This source estimation reveals the contributions of multiple sources to scalp EEG signals within a single cortical map. Figure 8 displays the EEG signal source estimation for the same action in both datasets, with a time interval of 250 ms spanning from −0.5 to 1 s. Figure 8A corresponds to Dataset I, while Figure 8B corresponds to Dataset II. This visualization is independent of TSPNet. The routines from the toolbox (Tadel et al., 2011) were employed to compute the inverse solutions for this visualization. The toolbox is open-source and available for free download at “https://github.com/aojeda/headModel.” As demonstrated in Figure 8, specific areas of the cerebral cortex become activated during motor imagination, resulting in corresponding changes in EEG signals.

#### 3.5.2 Feature visualization based on signal occlusion frequency

**Figure 9:**
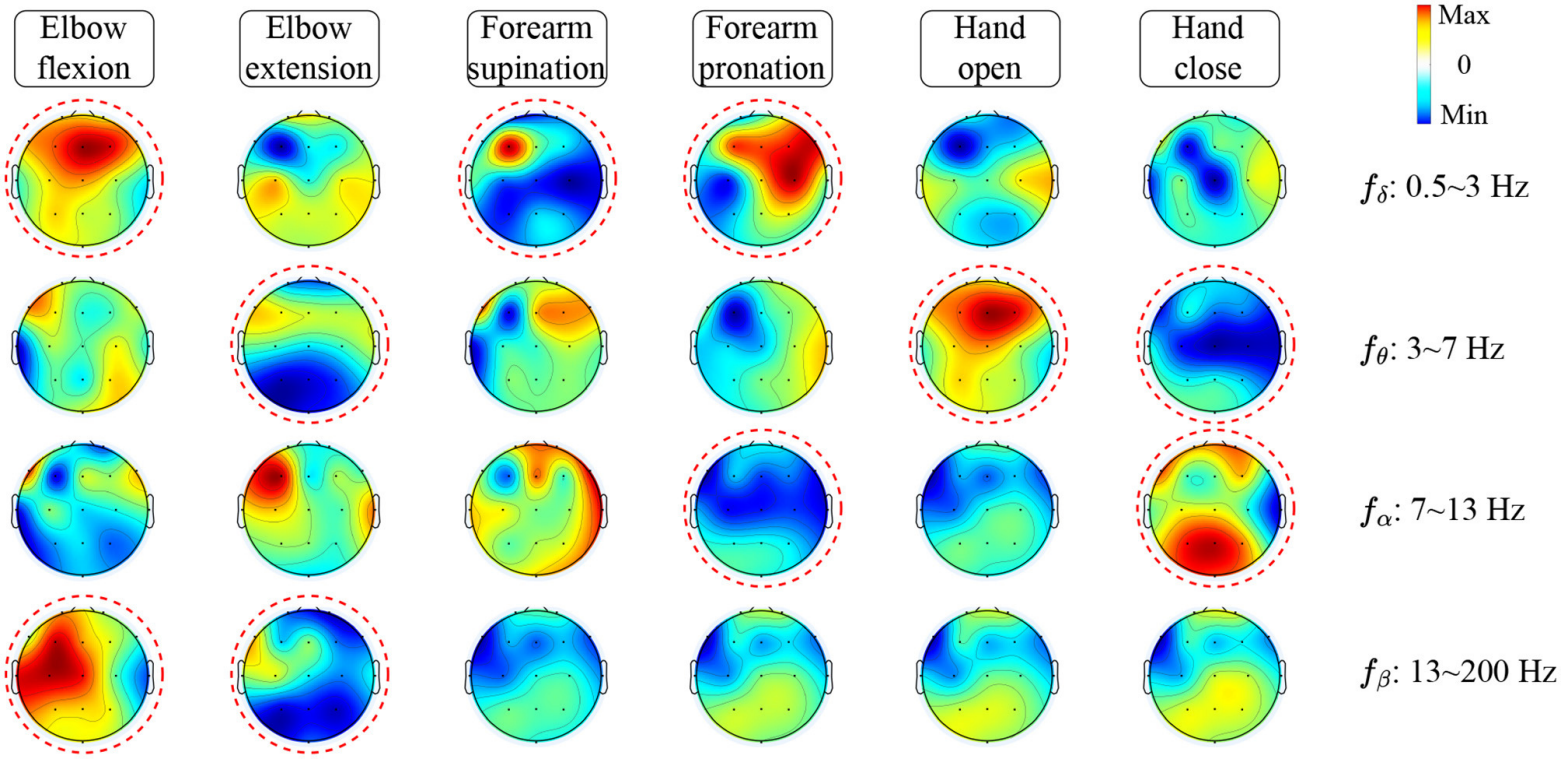
Feature visualization based on signal occlusion frequency.

To investigate how TSPNet can successfully decode information from EEG signals, Algorithm 1 is utilized to visualize the features extracted from TSPNet, and the results are presented in Figure 9. The red circles in the Figure 9 indicate distinct classifier patterns that can be used for differentiation. It can be observed from Figure 9 that the movements *hand open* and *hand close* exhibit distinct classifier patterns in the frequency ranges θ: 3–7 Hz and α : 7–13 Hz. Similarly, the movements *elbow flexion* and *elbow extension* display distinctive patterns at δ : 0.5–3 Hz, θ: 3–7 Hz, and β: 13–200 Hz, while the movements *forearm supination* and *forearm pronation* feature unique classifier patterns at δ: 0.5–3 Hz and α: 7–13 Hz. These visualization results demonstrate that the proposed framework is capable of generating distinct classifier patterns for various upper limb motor imagery categories across different frequency bands in EEG signals.

#### 3.5.3 Feature visualization before and after TSPFE

**Figure 10:**
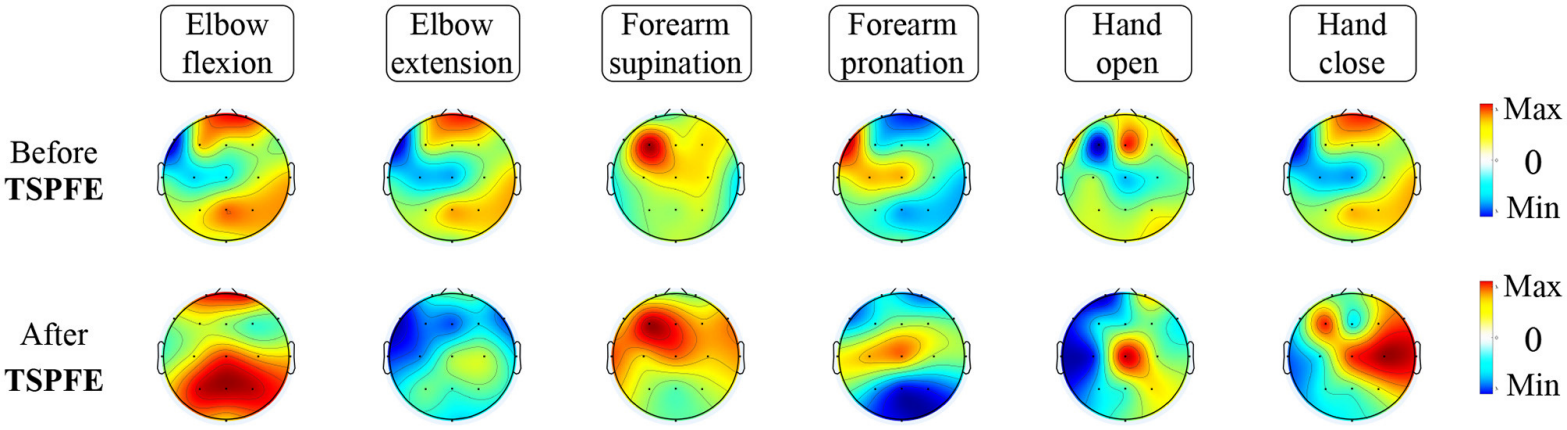
Feature visualization before and after TSPFE.

To elucidate the pivotal role of the TSPFE module in TSPNet, we present the transformation of feature maps before and after the TSPFE module into scalp topography maps in Figure 10. It can be observed that the features after the TSPFE module are more pronounced compared to those before the TSPFE. This is attributed to the fact that TSPFE module further extracts concurrent temporal and spatial features. The features before TSPFE undergo only time dimension feature extraction from the TDFE module and spatial feature extraction from the SDFE module. Combining the results of ablation experiments in Table 2, the absence of the TSPFE module results in a 24.3% accuracy decrease for TSPNet-w/o-TSPFE compared to TSPNet, while TSPNet-w/o-TDFE and TSPNet-w/o-SDFE experience decreases of 22.3 and 22.5%, respectively. This underscores the critical importance of TSPFE in TSPNet.

## 4 Discussion

In this work, we introduced TSPNet, a convolutional neural network classification model for motor imagery brain-computer interfaces. It enables the classification of six classes of upper limb movements based on motor imagery EEG signals. Our work provides a detailed explanation of its three constituent structures, TDFE, SDFE, and TSPFE. We conducted classification experiments on TSPNet using two datasets, comparing it with other deep learning methods [MSATNet (Hu et al., 2023), EEGSym (Perez-Velasco et al., 2022), DeepConvNet (Schirrmeister et al., 2017), EEGNet-8,2 (Lawhern et al., 2018)]. The experimental results demonstrate that our proposed TSPNet outperforms the compared methods in terms of classification accuracy. Additionally, results from the two-sample *t*-test indicate a significant difference in accuracy between TSPNet and the compared methods. Feature visualization results, as shown in Figure 10, suggest that the TSPFE module plays a crucial role in TSPNet. Before TSPFE, the TSFE module only convolves to extract time dimension features, while the SDFE module only convolves to extract spatial dimension features. TSPFE decouples the connection between time and spatial features, reducing feature redundancy. It utilizes a gating mechanism to optimize weight distribution, ultimately parallelizing time and spatial features. Furthermore, the proposed feature visualization algorithm based on signal occlusion frequency, qualitatively analyzes TSPNet's performance (as depicted in Figure 9), showing its ability to generate different classifier patterns for various classes across different frequency bands.

Compared to EEGNet (Lawhern et al., 2018) and MSATNet (Hu et al., 2023), TSPNet utilizes a signal frequency range of 0.01–200 Hz. This range is significantly broader than the signal frequency ranges used by EEGNet (0.1–40 Hz) and MSATNet (0.5–100 Hz). The qualitative analysis results of TSPNet using the feature visualization algorithm based on signal occlusion frequency indicate that TSPNet can generate different classifier patterns within the range of 0.1–200 Hz across various frequency bands. Additionally, TSPNet's signal sampling frequency of 500 Hz surpasses the sampling frequencies of EEGNet (128 Hz) and MSATNet (250 Hz). A higher sampling frequency results in a greater number of sampled points and more EEG information within a unit of time. It's worth noting that the TSPNet method proposed in this article demonstrates a higher accuracy (49.7% vs. 44%) in the motor imagery on Dataset II compared to the experimental results of Ofner et al. (2017) in movement execution. This difference can be attributed to several factors. Firstly, Ofner et al. utilized low-frequency signals (0.3–3 Hz), and the signal sampling frequency was 256 Hz, which is lower than the 500 Hz used in this paper. Secondly, and importantly, Ofner et al. employed a traditional approach involving feature extraction combined with machine learning classification patterns. The classification performance was highly dependent on the performance of the feature extraction algorithm. In contrast, TSPNet is an end-to-end deep learning model based on convolutional neural networks, where feature extraction and classification interact throughout the entire training process with shared weights, providing a distinct advantage in multi-class tasks.

In terms of limitations, despite the superior performance of the proposed TSPNet compared to other methods used in this paper, the accuracy in the six-class motor imagery task remains relatively low. Under the current research results, it is insufficient to generate precise and error-free control signals for the motion control of neural prosthetics or robotic arms. Several factors contribute to this limitation. Firstly, the intrinsic complexity and variability of EEG signals make achieving high decoding accuracy challenging. Secondly, EEG signals are generated by electrical potentials from different regions of the brain but are measured through electrodes placed on the scalp. Due to the conductivity and geometric properties of the head tissues, the recorded signals are spatially ambiguous and cannot accurately represent the potential neural sources. To address the current limitation of low classification accuracy, in future research, we will explore the integration of transfer learning into the classification of motor imagery EEG signals to enhance the performance of the classification model. Simultaneously, we will develop a continuous decoding strategy to further improve the classification accuracy of motor imagery tasks through multiple consecutive decoding steps.

## 5 Conclusion

In this article, the TSPNet is proposed to achieve intention recognition for multiclass upper limb motor imagery. Ablation studies demonstrate the necessity of each module in the proposed TSPNet. Our proposed TSPNet achieved a classification accuracy of 49.1% ± 0.043 in Dataset I and 49.7% ± 0.029 in Dataset II for 6 categories of upper limb motor imagery EEG signals. Comparison results with other deep learning methods demonstrate the superior performance of the TSPNet model. Subsequently, we introduce a feature visualization algorithm based on signal occlusion frequency to qualitatively analyze TSPNet. These visualization results demonstrate that the proposed TSPNet is capable of generating distinct classifier patterns for various upper limb motor imagery categories across different frequency bands in EEG signals. The results show that the proposed TSPNet can achieve intention recognition for multiple category upper limb motor imagery, which is of special significance in non-invasive BCI applications and provides the possibility to increase the degrees of freedom for devices controlled by BCI, such as robots, manipulators, or nerve rehabilitation devices.

## Data availability statement

The datasets presented in this study can be found in online repositories. The names of the repository/repositories and accession number(s) can be found at: https://dx.doi.org/10.21227/8qw6-f578.

## Author contributions

JB: Data curation, Methodology, Validation, Visualization, Writing—original draft. MC: Conceptualization, Project administration, Supervision, Writing—review & editing. GW: Data curation, Formal analysis, Writing—review & editing. XG: Data curation, Formal analysis, Writing—review & editing.
